# Pancreatic cancer revealed by a Sister Mary Joseph’s nodule

**DOI:** 10.11604/pamj.2020.36.113.22486

**Published:** 2020-06-22

**Authors:** Naoual El Omri, Fadwa Mekouar

**Affiliations:** 1 Internal Medicine B, Mohammed V Military Teaching Hospital, Sidi Mohamed Ben Abdellah University, Fes, Morocco

**Keywords:** Umbilical nodule, Sister Mary Joseph’s nodule, pancreatic cancer

## Image in medicine

Sister Mary Joseph’s nodule is a metastatic of a primary cancer, usually adenocarcinoma and associated with poor prognosis. Here we report the case of a 48-year-old man, admitted to the hospital with six months history of epigastric pain without vomiting or externalized hemorrhage and without transit disorders in a context of alteration of the general state with weight loss. Clinical examination showed epigastric sensitivity with a painful umbilical nodule, firm and irregular (A). Laboratory tests revealed a cholestasis. His computed tomography (CT) of the abdomen showed a mass lesion at the tail of the pancreas measuring 45 mm enclosing the splenic artery with secondary ganglionic, hepatic, bone and peritoneal localization (B, C). The umbilical lesion biopsies revealed metastasis of the pancreatic adenocarcinoma (D). The patient received palliative chemotherapy.

**Figure 1:**
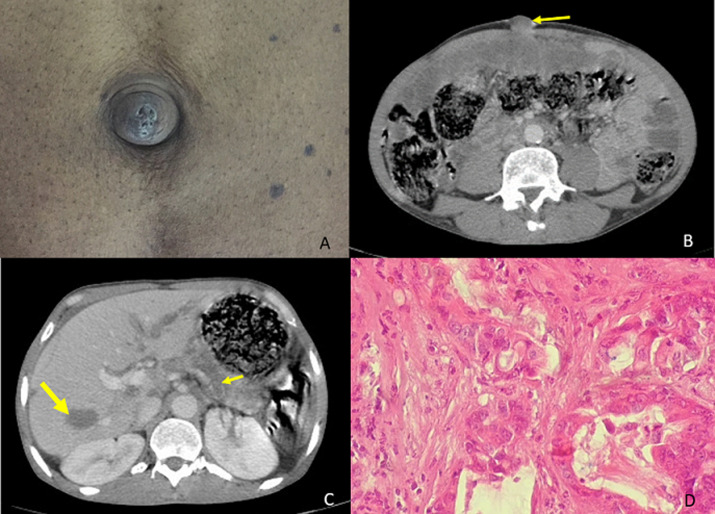
(A) sister Mary Joseph´s umbilical nodule; (B) the cross-sectional CT image of the umbilicus showing the umbilical nodule (arrow); (C) mass lesion at tail of the pancreas enclosing the splenic artery (small arrow) with secondary hepatic localization (big arrow); (D) picture HEx100: moderately differentiated adenocarcinoma with a mucosal colloid component whose immunohistochemical profile is consistent with a bilio-pancreatic origin

